# Measurement bone mineral density (BMD) of patients with beta thalassemia

**DOI:** 10.1016/j.dib.2018.05.120

**Published:** 2018-05-24

**Authors:** Sakineh Abbasi, Jafar Fatahi Asl, Leila Moein Zadeh, Mohammad Mirdoraghi

**Affiliations:** a Department of Laboratory Science, Tehran University of Medical Sciences, Tehran, Iran; b School of Para medicine, Department of Radiologic Technology, Ahvaz Jundishapur University of Medical Sciences, Ahvaz, Iran; c Thalassemia ward of Shafa hospital in Ahvaz, M.Sc in Hematology, Ahvaz, Iran; d Committee of Student Researches, Ahvaz Jundishapur University of Medical Sciences, Ahvaz, Iran

**Keywords:** Beta thalassemia, Osteoporosis, Bone mineral density, *Z*-Score, Ahvaz

## Abstract

The aim of the study is determining bone mineral density (BMD) of Patients with beta thalassemia in order to find the prevalence and related factors on the conditions. *Z*-Score of femoral neck and lumbar vertebrae were reported comparing normal matched subjects. Age and bone mineral density were significantly correlated. Moreover, the disease had significantly higher severity in men than in women. A negative significant correlation was detected between BMD and the mean of hematocrit in the last 5 years. There was significant differences between sex hormone and bone density. A significant correlation between hydroxy urea and BMD were found. A significant relationship between the use of bisphosphonates and bone density were found. Osteopenia and osteoporosis were highly prevalent in our participants. Therefore, regular tests are required to examine bone mineral density in these patients. Furthermore, the exact effect of age on bone mineral density need to be clarified.

**Specifications Table**

**Table:** 

Subject area	Medical Physics
More specific subject area	Describe the bone mineral density (BMD) of patients with beta thalassemia
Type of data	Tables
How data was acquired	To calculate the bone mineral density, All medical records of patients older than 15 years were reviewed. Patients demography were extracted, the results of the BMD and *Z*-Score of femoral neck and lumbar vertebrae were reported comparing normal matched subjects. *Z*-Scores <−1, −1 to −2.5, and bigger than −2.5 were considered as Normal, osteopenia and osteoporotic respectively.
Data format	Raw, Analyzed
Experimental factors	The mentioned parameters above, in abstract section, were analyzed according to the standards for *Z*-Score.
Experimental features	The *Z*-Scores were determined.
Data source location	Ahvaz province, Iran.
Data accessibility	The data are available whit this article

**Value of the data**

• This study showed bone mineral density reduced with increasing age (*p*<0.03).
• Measured BMD had a better status in the femoral region than in the lumbar region.
• Since the patients were in puberty, there was a significant correlation between the use of sex hormones and BMD.
• The bone mineral density increased in patients who taking bisphosphonates.

## 1 Data

The participants included 81 male and 103 female patients with transfusion-dependent thalassemia (β-thalassemia). As seen in Table 1, the mean age of the participants was 23.4±8.0 years (range: 15–31 years). Based on the *Z*-scores at the lumbar spine, 198 patients (82%) had low BMD (*Z* ≤−2) and 40 (18%) had normal BMD (*Z* ≥−1). Meanwhile, low BMD in the neck of the femur was only seen in 18% of the participants. A significant correlation between age and bone mineral density were found (*P*<0.03). Per year to 2.8 percent of bone mineral density scores are low. The severity of the disease was higher in men than in women (*P*<00.02). A negative significant correlation was detected between BMD and the mean of hematocrit in the last 5 years (*P*<0.005). There was significant differences between Sex hormone and bone density (*P*<0.02). The significant relationship between Hydroxyl urea consumption and bone mineral density was seen (*P*<00.01). A significant relationship between the use of bisphosphonates and bone density exists so that the bone mineral density in patients taking bisphosphonates have been increased (*P*<0.02). The patients who used calcium+vitamin D had no significant difference with the others in terms of BMD. Moreover, no significant correlation was found between the use of calcitriol and BMD.

**Table 1:** Demographic features of patients (confidence interval=95%).

**Demographic features of patients**	**Time consuming(year)**
Calcium+Vitamin D of consuming time to first measurement	1/8±2/4
Calcium+Vitamin D of consuming time to the second measurement time	6/3±3
Calcium+Vitamin D starting age	22±7
Age of onset of sex hormones	15±6
Duration of sex hormones to first measure	6/3±4
Duration of sex hormones to the second measure	9/6±3
Age of onset of Calcitriol	21±7
Calcitriol duration of the first measure	2±3
Calcitriol use to measure a second time	6±3
Age starting bisphosphonate	22±7
bisphosphonate use to measure of the first time	1±2
bisphosphonate use to measure a second time	2/3±2
Age starting hydroxyurea	16/5±6
hydroxyurea use to measure of the first time	5/9±3/6
hydroxyurea use to measure a second time	6/2±3/2

## 2 Experimental design, materials and methods

A total of 216 patients (age>15 years) with transfusion-dependent thalassemia who visited Shafa Hospital (Ahwaz, Iran) participated in this prospective study. Their BMD had been annually monitored from age 10. The bone density in 216 patients (81 males and 103 females) who are suffering from thalassemia major, by Dual-Energy X-ray Absorptiometry.

(Manufactured by Lunar company of USA) to be measured [1], [2]. The patients׳ demographic characteristics, average five-year hematocrit level (2008–2013), and history of medications were extracted from their records. DEXA was then performed twice to measure BMD in the femur and lumbar region [3], [4]. To reduce measurement errors in each region measured three times mineral density and an average is taken (Table 2).

**Table 2:** The average of *Z*-Score before and after intake of drug (confidence interval=95%).

**Demographic features of patients**	**The average of *Z*-Score in the first time**	**The average of *Z*-Score in the second time**	***P*-value**
Calcium+Vitamin D	−2/33±0/78	–2/43±0/97	*P*>0.05
sex hormones	−2/41±0/26	−1/91±0/11	*P*<0.02
Calcitriol duration	−1/44±0/18	−1/98±0/36	*P*>0.05
bisphosphonate	−2/45±0/88	−1/88±0/22	*P*<0.02
Hydroxy urea use to measure of the first time	−2/59±0/96	−2/50±0/24	*P*<00.01

To view the impact of drugs on patients before and after drug consumption *Z* and *T* score were measured before and after drug consumption. In measuring the density by Dual-Energy X-ray Absorptiometry each person has three criteria: first index (*Z*): actually indicates Compare between patient׳s bone density and bone density persons are same age and gender. The second index (*T*): the comparison between the patient׳s bone density and bone density persons are same age [5], [6], [7].

*Z*-scores were obtained from BMD measurements and used to decide whether the patients had osteopenia or osteoporosis. Data analysis was performed with software SPSS 20 and the effect of drugs was studied by Wilcoxon test.
