# Supplementary material for: Measurement bone mineral density (BMD) of patients with beta thalassemia
Source: Data Brief. 2018 May 24;19:1021–4. doi: 10.1016/j.dib.2018.05.120 (PMC5998177; doi:10.1016/j.dib.2018.05.120)
Supplement: Supplementary file 1 — Supplementary material [file mmc1.docx]

**Conflict of Interest:**

None to declare.
